# Human Adipose Tissue-Derived Mesenchymal Stem Cells Attenuate Atopic Dermatitis by Regulating the Expression of MIP-2, miR-122a-SOCS1 Axis, and Th1/Th2 Responses

**DOI:** 10.3389/fphar.2018.01175

**Published:** 2018-11-06

**Authors:** Misun Kim, Sung-Hoon Lee, Youngmi Kim, Yoojung Kwon, Yeongseo Park, Hong-Ki Lee, Hyun Suk Jung, Dooil Jeoung

**Affiliations:** ^1^Department of Biochemistry, Kangwon National University, Chunchon, South Korea; ^2^Biotechnology Institute, EHL-BIO Co., Ltd., Uiwang, South Korea

**Keywords:** adipose tissue-derived stem cells, atopic dermatitis, targets of stem cells, cellular interactions, MIP-2, miR-122a-5p, SOCS1

## Abstract

The objective of this study was to investigate the effect of human adipose tissue-derived mesenchymal stem cells (AdMSCs) on atopic dermatitis (AD) in the BALB/c mouse model. The AdMSCs attenuated clinical symptoms associated with AD, decreased numbers of degranulated mast cells (MCs), IgE level, amount of histamine released, and prostaglandin E2 level. Atopic dermatitis increased the expression levels of cytokines/chemokines, such as interleukin-5 (IL-5), macrophage inflammatory protein-1ß (MIP-1ß), MIP-2, chemokine (C-C motif) ligand 5 (CCL5), and IL-17, in BALB/c mouse. The AdMSCs showed decreased expression levels of these cytokines in the mouse model of AD. *In vivo* downregulation of MIP-2 attenuated the clinical symptoms associated with AD. Atopic dermatitis increased the expression levels of hallmarks of allergic inflammation, induced interactions of Fc𝜀RIβ with histone deacetylase 3 (HDAC3) and Lyn, increased ß-hexosaminidase activity, increased serum IgE level, and increased the amount of histamine released in an MIP-2-dependent manner. Downregulation of MIP-2 increased the levels of several miRNAs, including miR-122a-5p. Mouse miR-122a-5p mimic inhibited AD, while suppressor of cytokine signaling 1 (SOCS1), a predicted downstream target of miR-122a-5p, was required for AD. The downregulation of SOCS1 decreased the expression levels of MIP-2 and chemokine (C-X-C motif) ligand 13 (CXCL13) in the mouse model of AD. The downregulation of CXCL13 attenuated AD and allergic inflammation such as passive cutaneous anaphylaxis. The role of T cell transcription factors in AD was also investigated. Atopic dermatitis increased the expression levels of T-bet and GATA-3 [transcription factors of T-helper 1 (Th1) and T-helper 2 (Th2) cells, respectively] but decreased the expression of Foxp3, a transcription factor of regulatory T (Treg) cells, in an SOCS1-dependent manner. In addition to this, miR-122a-5p mimic also prevented AD from regulating the expression of T-bet, GATA-3, and Foxp3. Atopic dermatitis increased the expression of cluster of differentiation 163 (CD163), a marker of M2 macrophages, but decreased the expression of inducible nitric oxide synthase (iNOS), a marker of M1 macrophages. Additionally, SOCS1 and miR-122a-5p mimic regulated the expression of CD163 and iNOS in the mouse model of AD. Experiments employing conditioned medium showed interactions between MCs and macrophages in AD. The conditioned medium of AdMSCs, but not the conditioned medium of human dermal fibroblasts, negatively inhibited the features of allergic inflammation. In summary, we investigated the anti-atopic effects of AdMSCs, identified targets of AdMSCs, and determined the underlying mechanism for the anti-atopic effects of AdMSCs.

## Introduction

Mesenchymal stem cells (MSCs) are multipotent stem cells that can be isolated from various tissues, including bone marrow, umbilical cord and adipose tissue. They interact with both innate and adaptive immune systems, resulting in a suppressive effect on the activation of immune cells, including T cells, B cells, dendritic cells, and natural killer (NK) cells (Asari et al., 2009; Prigione et al., 2009). Mesenchymal stem cells can ameliorate allergic diseases such as asthma, rhinitis, and dermatitis (Nemeth et al., 2010; Kavanagh and Mahon, 2011; Kapoor et al., 2012), and MSCs suppress chronic asthmatic changes in male rats (Ahmadi et al., 2016). Intratracheally administered MSCs can attenuate inflammation and promote tissue homeostasis in the course of allergic asthma (Urbanek et al., 2016). In addition, MSCs can change microRNA (miRNA) expression profile in airway allergic inflammation (Tang et al., 2016), suppress lung inflammation and airway remodeling via PI3K/Akt signaling pathway in rat asthma model (Lin et al., 2015), and ameliorate allergic airway inflammation by blunting the amplification of epithelial-derived inflammatory cytokines (Duong et al., 2015). The overexpression of C-X-C motif chemokine receptor 5 (CXCR5) is known to intensify the immunomodulatory effects of MSCs, and it decreases allergic contact dermatitis (ACD) (Zhang et al., 2017).

Human umbilical cord blood-derived MSCs (hUC-MSCs) are known to exhibit protective effects against AD and suppress infiltration and degranulation of mast cells (MCs) (Kim et al., 2015). Transforming growth factor (TGF-β1) production from hUC-MSCs in response to interleukin-4 (IL-4) contributes to the attenuation of MC degranulation by downregulating Fc𝜀RI expression in MCs (Kim et al., 2015). Adipose tissue-derived mesenchymal stem cells (AdMSCs) contribute to the self-limiting course of ACD by decreasing the expression of interferon-γ (IFN-γ) (Kikuchi et al., 2017). They can reduce gross and histological signatures of AD induced by *Dermatophagoides farinae*, decrease serum IgE level, and suppress MC degranulation (Shin et al., 2017).

Although previous reports have suggested the roles of AdMSCs in treating allergic diseases, their efficacy and mechanisms in treating AD have not been extensively investigated yet. In this study, we showed the anti-atopic effect of AdMSCs and identified macrophage inflammatory protein-2 (MIP-2) as a target of AdMSCs in a mouse model of AD. Macrophage inflammatory protein-2 acted as a negative regulator of miR-122a-5p. Underlying mechanisms of the anti-atopic effect of AdMSCs were also identified by determining the effects of MIP-2, miR-122a-5p-suppressor of cytokine signaling 1 (SOCS1) negative feedback loop on the clinical symptoms, expression levels of hallmarks, immune responses, and cellular interactions accompanied by AD. A conditioned medium of AdMSCs was employed to investigate the mechanism of the anti-atopic effect of AdMSCs in relation with cellular interactions during AD. Our study may offer a valuable strategy for the development of anti-atopic therapy.

## Materials and Methods

### Animals and Materials

Female BALB/c mice that were five weeks old were purchased from Nara Biotech (Seoul, South Korea) and were group housed under specific pathogen-free conditions in the animal facility of the Kangwon National University. All animal experiments were approved by the Institutional Animal Care and Use Committee (IACUC) of the Kangwon National University (KIACUC-160329-2). Acetone and 2, 4-dinitrochlorobenzene (DNCB) were purchased from Sigma-Aldrich (St. Louis, MO, United States). All other chemicals used in this study were also purchased from Sigma-Aldrich (St. Louis, MO, United States). The following antibodies were obtained from Santa Cruz Biotechnology (Dallas, TX, United States): MIP-2, histone deacetylase 3 (HDAC3), transglutaminase II (TGaseII), cyclooxygenase-2 (COX-2), monocyte chemoattractant protein 1 (MCP1), Lyn, SOCS1, Fc𝜀RIβ, and actin. Anti-mouse and anti-rabbit IgG horseradish peroxidase (HRP)-conjugated antibodies were purchased from Thermo Pierce (Rockford, IL, United States). The jetPRIME transfection reagent was purchased from Polyplus (NY, United States). The miRNA mimic and siRNAs were purchased from Bioneer Company (Daejeon, South Korea). The MIP-2 siRNA [5′-GAGUUGGGAACUAGCUACA-3′ (sense) and 5′-UGUAGCUAGUUCCCAACUC-3′ (antisense)] and scrambled siRNA [5′-GGTACGTCTCGAAGATAGA-3′ (sense) and 5′- GCTATCGCCCATTGCTAAT-3′ (antisense)]; SOCS1 siRNA [5′-GUGACUACCUGAGUUCCUU-3′ (sense) and 5′-AAGGAACUCAGGUAGUCAC-3′ (antisense)]; and CXCL13 siRNA [5′-GAGGAAUGAAAAACCUACA-3′ (sense) and 5′-UGUAGGUUUUUCAUUCCUC-3′ (antisense)] were used. Primers used in this study were commercially synthesized by Bioneer Company (Daejeon, South Korea).

### Isolation and Culture of Human Adipose Tissue-Derived Mesenchymal Stem Cells

The AdMSCs were isolated from human fat tissue obtained by liposuction (Shin et al., 2017). Fat tissue was washed with α-minimum essential medium (α-MEM; Gibco, Grand Island, NY, United States) and was digested with an 0.1% collagenase (type I, Gibco, Grand Island, NY, United States) solution at 37°C for 30 min. Cell pellet obtained by centrifugation at 2,000 rpm for 10 min was suspended in α-MEM containing 8% fetal bovine serum (Gibco, Mulgrave Victoria, Australia) and filtrated with 100 μm nylon mesh. Cell suspension was incubated at 37°C in 5% CO_2_ for 24 h, and unbound cells were removed by washing. Cells were passaged five times by the TrypLE^TM^ (Gibco Grand Island, NY, United States) method, and surface cluster of differentiation (CD) markers of AdMSCs were analyzed by FACS (FACSVerse, BD). All AdMSC banking was conducted in accordance with guidelines approved by the Korea National Institute for Bioethics Policy’s IRB (IRB No. P01-201511-31-004).

### Culture of Human Dermal Fibroblasts

Human primary dermal fibroblasts were purchased (HDFa lot #1780051, Gibco, Grand Island, NY, United States) and expanded in Dulbecco’s modified eagle medium (DMEM; Gibco, Grand Island, NY, United States) containing 8% fetal bovine serum (Gibco, Mulgrave Victoria, Australia) at 37°C with 5% CO_2_.

### Induction of Atopic Dermatitis in BALB/c Mice

**FIGURE 1:**
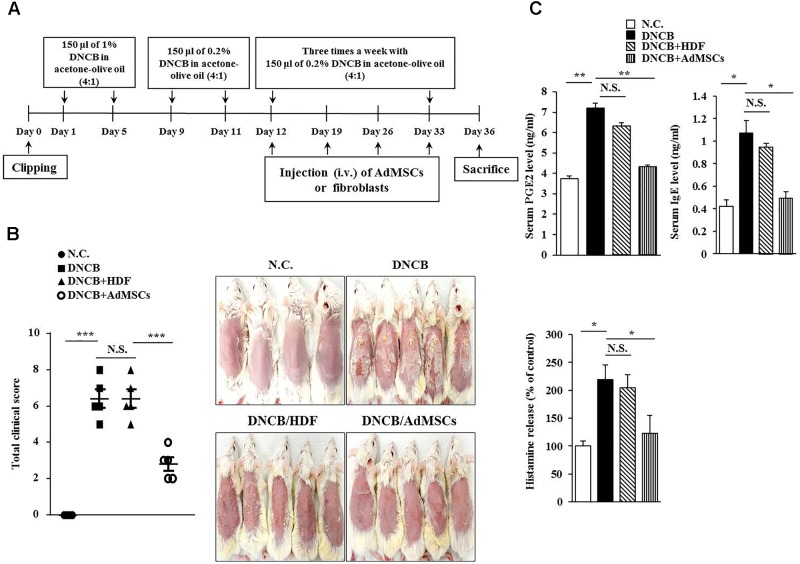
Human adipose tissue-derived mesenchymal stem cells (AdMSCs) attenuate the clinical symptoms associated with atopic dermatitis (AD). **(A)** 2, 4-dinitro chlorobenzene (DNCB) was employed to induce AD in BALB/c mice. Human dermal fibroblasts (HDFs) or AdMSCs were intravenously injected once a week. **(B)** At the indicated day (day 36) after the induction of AD, clinical scores of BALB/c mice of each experimental group were determined. ^∗∗∗^*p* < 0.0005. N.C., negative control; N.S., not significant. **(C)** Sera of BALB/c mice of each experimental group were employed for the determination of prostaglandin E2 (PGE2) level, IgE level, and the amount of histamine released. ^∗^*p* < 0.05; ^∗∗^*p* < 0.005.

Symptoms of atopic dermatitis (AD) were induced by using DNCB, as previously described (Kwon et al., 2010), with minor modifications. Briefly, the upper backs of the mice were shaved with a clipper. After 24 h, 150 μl of 1% DNCB in acetone: olive oil mixture (4:1 vol/vol) was topically applied on days 1 through 5. Later, the same dose of 0.2% DNCB was applied three times a week for 4 weeks. Human AdMSCs [1 × 10^6^ cells in 100 μl phosphate buffered saline (PBS)] were injected intravenously on days 12 and 23 of the timeline (Figure 1A). The cell control group was injected with human dermal fibroblast (HDF). Dermatitis scores of 0 (none), 1 (mild), 2 (moderate), and 3 (severe) were given for each of the four symptoms: dryness, excoriation, erosion, and erythema and edema. The sum of the individual scores was used as the clinical severity. After sacrifice on day 26, serum and skin samples were collected and stored at -80°C to detect the concentration of total IgE, prostaglandin E2 (PGE2), and the amount of histamine released or to evaluate histopathological lesions. The experimental design is described in Figure 1A. To examine the effect of MIP-2 on AD, mice were intravenously injected with MIP-2 siRNA (100 nM) or scrambled siRNA (100 nM) for a total of seven times as described in the timeline (Figure 4A). To examine the effect of SOCS1 on AD, mice were intravenously injected with SOCS1 siRNA (100 nM) or scrambled siRNA (100 nM) for a total of five times as described in the timeline (Figure 6B). To examine the effect of miR-122a-5p on AD, mice were intravenously injected with control mimic (100 nM) or miR-122a-5p mimic (100 nM) for a total of six times as described in the timeline (Figure 8A).

### Histological Examination

To evaluate skin thickening and cellular infiltration, skin samples were collected, fixed with 10% neutral buffered formalin, and embedded in paraffin. Sections (5 μm thickness) were prepared and stained with haemotoxylin and eosin (H&E) or toluidine blue for leukocyte infiltration or MC infiltration and degranulation, respectively.

### The Levels of IgE, PGE2, and Histamine Release

The levels of IgE, PGE2, and the amount of histamine released were measured according to the manufacturer’s instruction using the commercially available ELISA kit (Abcam, United Kingdom). Reaction product was measured calorimetrically with a microplate reader. Histamine release assays employing sera of BALB/c mice were performed according to the manufacturer’s instruction (Enzo, United States).

### Cytokine Array

Expression levels of cytokine/chemokines were determined by using a Proteom ProfilerTM Mouse Cytokine Array Kit (R&D Systems, MN, United States) according to the manufacturer’s instructions. Blood serum collected from the BALB/c mouse of each experimental group was incubated with an array containing 40 mouse cytokine and chemokine specific antibodies. Relative cytokine intensities were normalized in comparison with control spots on the same membrane.

### miRNA Array

The miRNA expression analysis in AdMSCs was performed by using miRNA Array III (Signosis, CA, United States) following the manufacturer’s instructions. Briefly, 5 mg of total miRNA was annealed to an oligonucleotide primer mix and hybridized to 132 miRNA oligonucleotide probes. Streptavidin-HRP chemiluminescence was used for the detection of miRNA expression.

### miRNA Extraction and Quantitative Real-Time PCR

Total miRNA was isolated with the miRNeasy Micro Kit (Qiagen, CA, United States) following the standard protocol from the manufacturer. The extracted miRNA was reverse transcribed using a miScript II RT Kit (Qiagen, CA, United States) with universal RT primer. The expression level of miR-122a-5p was quantified with SYBR Green Master Mix (Qiagen, CA, United States) using a miRNA-specific forward primer and universal reverse primer. Relative expression of miRNA was calculated using the 2^-ΔΔCT^ method (ΔCT = CT_miR_ -CT_reference_). The U6 small nuclear RNA was used as an endogenous control for data normalization. The expression levels of MIP-2, SOCS1, and T-helper 1 (Th1)/ T-helper 2 (Th2) cytokines were also quantified by quantitative real-time PCR (qRT-PCR) analysis.

### miRNA Target Analysis

Genes that contain the miR-binding site(s) in the untranslated region (UTR) were obtained using the TargetScan^1,2,3^ program.

### Cell Culture

Isolation of macrophages was performed according to the standard procedures with slight modifications (Eom et al., 2014a). Isolation of MCs was performed according to the standard procedures with slight modifications (Eom et al., 2014b). Rat basophilic leukemia (RBL2H3) cells were obtained from the Korea Cell Line Bank (Seoul, South Korea). Cells were grown in DMEM containing heat-inactivated fetal bovine serum, 2 mM L-glutamine, 100 U/ml penicillin, and 100 μg/ml streptomycin (Invitrogen). Cultures were maintained in 5% CO_2_ at 37°C.

### Western Blot and Immunoprecipitation Analysis

Western blot and immunoprecipitation analyses were performed according to the standard procedures (Eom et al., 2014a). Tissue homogenates obtained from frozen samples were lysed in a radioimmunoprecipitation assay (RIPA) buffer containing 50 mM Tris-HCl, pH 8.0, 150 mM NaCl, 0.5% sodium deoxycholate, 0.1% sodium dodecyl sulfate, 1 mM NaF, 1 mM Na3VO4, 1 mM leupeptin, 20 μg/ml aprotinin, and 1% Nonidet P-40. Protein content of the supernatant was quantified using the Bio-Rad protein assay reagent. Equal amounts of proteins were separated on 10% SDS-polyacrylamide gels, transferred to PVDF membrane, and then exposed to the appropriate antibodies. For immunoprecipitation, 100 μg of protein was incubated with anti-Fc𝜀RIβ antibody (2 μg/ml), and immunocomplexes were precipitated using protein A/G Sepharose and analyzed by western blot.

### β-Hexosaminidase Activity Assays

β-hexosaminidase activity assays were performed according to standard procedures as described previously (Noh et al., 2017).

### Immunohistochemical Staining

Immunohistochemical staining of tissues was performed using an established avidin-biotin detection method (Vectastain ABC kit, Vector Laboratories Inc., Burlingame, CA, United States). 4–6 μm-thick sections of the paraffin-embedded tissue blocks were cut, mounted on positively charged glass slides, and dried in an oven at 56°C for 30 min. The sections were deparaffinized in xylene and then rehydrated in graded ethanol and water. Endogenous peroxidase was blocked by incubation in 3% (v/v) hydrogen peroxide for 15 min. Antigen retrieval was accomplished by pretreatment of the sections with citrate buffer at pH 6.0 for 20 min at 56°C in a microwave oven, and then the sections were allowed to cool for 30 min. Nonspecific endogenous protein binding was blocked using 1% bovine serum albumin (BSA). The sections were then incubated with primary antibodies overnight at 4°C. The following primary antibodies were used: anti-SOCS1 (1:100, Santa Cruz Biotechnology); anti-MIP-2 (1:100, Invitrogen); anti-CXCL13 (1:250, R&D Systems); anti-CD163 (1: 700, Abchem); and anti-inducible nitric oxide synthase (iNOS) (1: 500, Santa Cruz Biotechnology). After washing, biotinylated secondary antibodies were applied at 1:100 or 1:200 dilutions for 1 h. Color was developed with diaminobenzidine (Vector Laboratories, Inc.). Sections were counterstained with Mayer’s hematoxylin. The sections incubated without primary antibody served as controls. To visualize tissue MCs, the sections were stained with 0.1% toluidine blue (Sigma) in 0.1 N HCl for 15 min.

### Passive Cutaneous Anaphylaxis (PCA)

The BALB/c mice were sensitized with an intradermal injection of 2, 4-dinitrophenol (DNP)-specific IgE (0.5 μg/kg). After 24 h, the mice were challenged with an intravenous injection of DNP-human serum albumin (HSA) (250 μg/kg) and 2% (v/v) Evans blue solution. After 30 min of DNP-HSA challenge, the mice were euthanized, and the 2% (v/v) Evans blue dye was extracted from each dissected ear in 700 μl of acetone/water (7:3) overnight. The absorbance of Evans blue in these extracts was measured with a spectrophotometer at 620 nm. To determine the effect of CXCL13 on the PCA, BALB/c mice were given an intradermal injection of IgE (0.5 μg/kg) and intravenous injection of the indicated siRNA (each at 100 nM). The next day, BALB/c mice were given an intravenous injection of PBS or DNP-HSA (250 μg/kg) along with 2% (v/v) Evans blue solution for determining the extent of vascular permeability accompanied by PCA. Later, 1 h after the injection of Evans blue solution, the dye was eluted from the ear in 700 μl of formamide at 63°C. The absorbance was measured at 620 nm. To determine the effect of the conditioned medium on PCA, BALB/c mice were given an intradermal injection of DNP-specific IgE (0.5 μg/kg) and an intravenous injection of the conditioned medium of AdMSCs or HDFs (each at 200 μl/mouse). The following day, BALB/c mice were administered an intravenous injection of PBS or DNP-HSA (250 μg/kg) along with 2% (v/v) Evans blue solution for determining the extent of vascular permeability accompanied by PCA.

### Statistical Analysis

Data were analyzed and graphed using the GraphPad Prism statistics program (GraphPad Prism software). Results are presented as means ± SE. Statistical analysis was performed using *t*-tests with differences between means considered significant when *p* < 0.05.

## Results

### Human Adipose Tissue-Derived Mesenchymal Stem Cells Inhibit Features of Atopic Dermatitis

**FIGURE 2:**
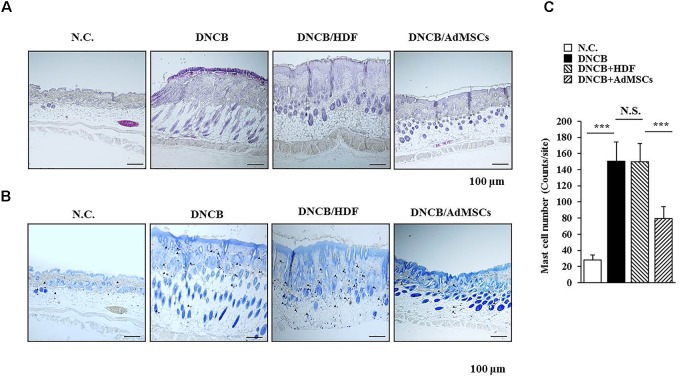
AdMSCs inhibit features of AD. **(A)** Skin tissues of BALB/c mice of each experimental group were isolated at the time of sacrifice and were subjected to hematoxylin & eosin (H&E) staining. **(B,C)** Same as **(A)** except that toluidine blue staining was performed to determine the number of degranulated mast cells. ^∗∗∗^*p* < 0.0005. Closed triangle represents degranulated mast cells. Scale bars represent 100 μm.

Several reports have shown the anti-allergic effects of MSCs (Nemeth et al., 2010; Kavanagh and Mahon, 2011; Kapoor et al., 2012; Ahmadi et al., 2016). This led us to hypothesize that MSCs might be able to regulate AD. We examined the effect of human AdMSCs on AD. For this, AD in BALB/c mouse was induced by DNCB. The BALB/c mice were treated with 1% (v/v) DNCB on days 1 through 5 (Figure 1A). These mice were treated again with 0.2% (v/v) DNCB on days 9 through 11. To examine the effect of AdMSCs on AD, AdMSCs (2 × 10^6^ cells) were injected intravenously at day 12 when AD was fully induced. The AdMSCs were injected intravenously once a week after the induction of AD. The HDFs were used as negative controls. The AdMSCs attenuated the clinical symptoms associated with AD (Figures 1B,C). The HDFs failed to affect the clinical symptoms associated with AD (Figure 1B). Additionally, AdMSCs prevented DNCB from increasing the levels of PGE2, IgE, and the amount of histamine released in the sera of BALB/c mice (Figure 1C). Staining with H&E showed that epidermal hyperplasia and lymphocyte infiltration induced by AD were attenuated by AdMSCs, but not by HDFs (Figure 2A). Toluidine blue staining showed that AdMSCs, but not HDFs, decreased the number of degranulated MCs in the mouse model of AD (Figures 2B,C). Taken together, these results suggest that AdMSCs can attenuate the clinical symptoms associated with AD. However, the mechanism for the anti-atopic effects of AdMSCs remains unknown.

### AdMSCs Exert Negative Effect on Increased Expression of Cytokines in Mouse Model of Atopic Dermatitis

**FIGURE 3:**
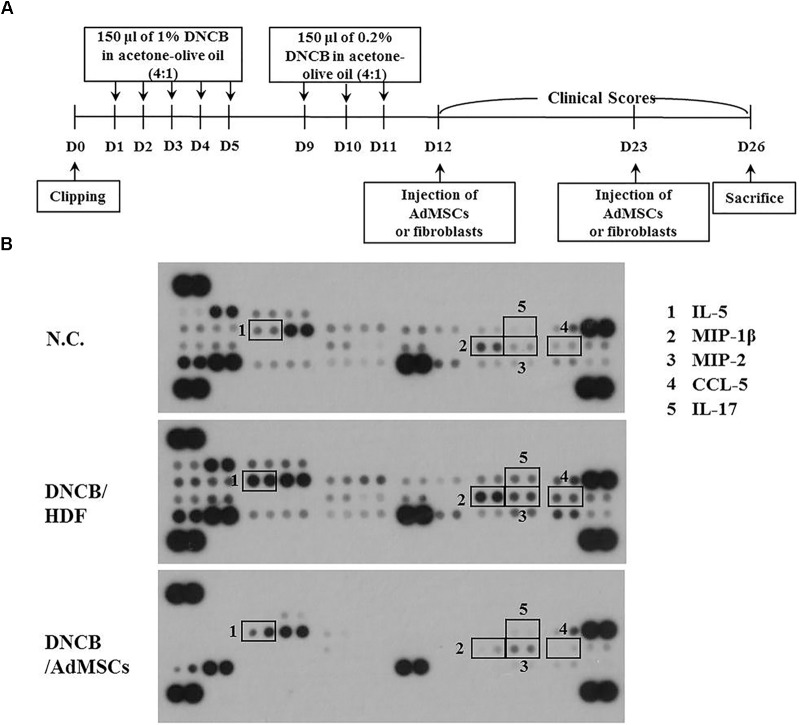
Identification of cytokines subjected to opposite regulations by AD and AdMSCs. **(A)** Shows the experimental time line to determine cytokines that mediate AD and serve as targets of AdMSCs. **(B)** Serum of BALB/c mouse of each experimental group was subjected to cytokine array analysis. Serum was obtained at the time of sacrifice.

Previous reports have suggested the roles of cytokines in anti-allergic effects of MSCs (Duong et al., 2015; Zhang et al., 2017). We hypothesized that AdMSCs could target cytokines to exert anti-atopic effects. For this, mouse model of AD was used (Figure 3A), and serum obtained from the BALB/c mouse of each experimental group was subjected to cytokine array analysis (Figure 3B). Atopic dermatitis increased the expression levels of IL-5, IL-17, chemokine (C-C motif) ligand 5 (CCL5), MIP-1ß, and MIP-2 in the serum of BALB/c mouse (Figure 3B). The AdMSCs, but not HDFs, prevented DNCB from increasing the expression of these cytokines (Figure 3B). Macrophage inflammatory protein-2 is necessary for AD induced by 1-Fluoro-2, 4-dinitrofluorobenzene in an Nc/Nga mouse model (Kim et al., 2010). Acute AD lesion shows a significant upregulation of Th2 promoting chemokines, such as CCL5 and CCL17 (Olivry et al., 2016). Taken together, these results suggest that AdMSCs may exert an anti-atopic effect by regulating the expression of inflammatory cytokines/chemokines such as MIP-2.

### MIP-2 Is Necessary for DNCB-Induced Atopic Dermatitis

**FIGURE 4:**
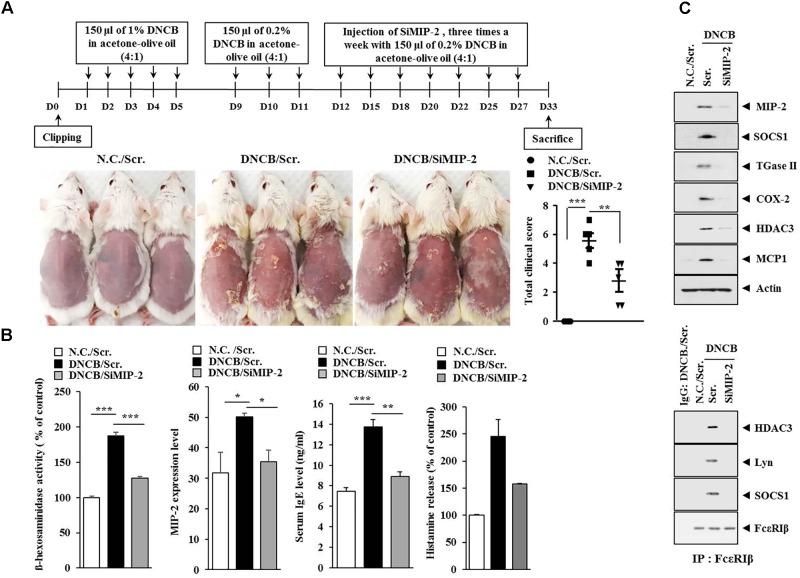
Macrophage inflammatory proten-2 (MIP-2) is necessary for AD. **(A)** Shows the experimental time line to determine the effect of MIP-2 on AD. Lower figure shows the effect of MIP-2 on clinical symptoms associated with AD. ^∗∗^*p* < 0.005; ^∗∗∗^*p* < 0.0005. **(B)** Skin tissue lysates were subjected to ß-hexosaminidase activity assay and qRT-PCR analysis. Serum IgE level and the amount of histamine released were also determined. ^∗^*p* < 0.05; ^∗∗^*p* < 0.005; ^∗∗∗^*p* < 0.0005. **(C)** Tissue lysates from the mice of each experimental group were subjected to western blot analysis and immunoprecipitation by employing the indicated antibody (2 μg/ml). Tissue lysates from BALB/c mouse injected with scrambled (scr.) following the induction of AD by DNCB were also immunoprecipitated with isotype-matched IgG antibody (2 μg/ml).

**FIGURE 5:**
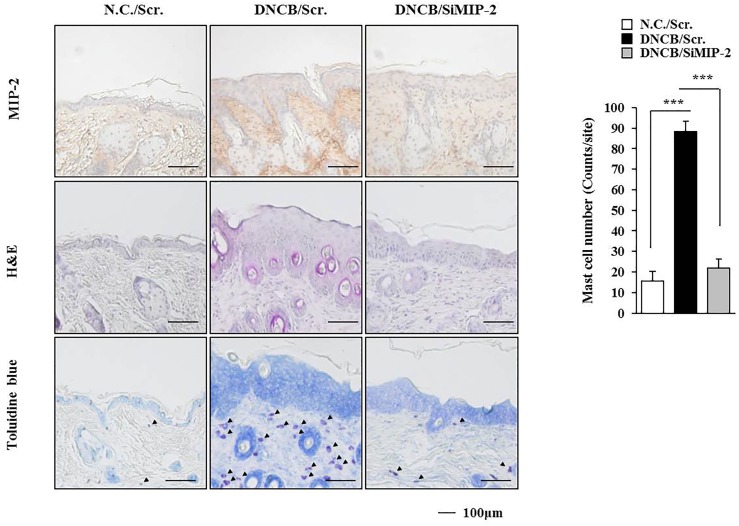
Induction of the features of AD occurs in an MIP-2-dependent manner. Skin tissues of BALB/c mice of each experimental group were isolated at the time of sacrifice and were subjected to immunohistochemical staining by employing anti-MIP-2 antibody (2 μg/ml) and H&E staining. Toluidine blue staining was also performed to determine the number of degranulated mast cells. Closed triangle represents degranulated mast cells. ^∗∗∗^*p* < 0.0005.

The level of MIP-2 was decreased by AdMSCs in the mouse model of AD (Figure 3B). The role of MIP-2 in AD has not been studied extensively when compared with cytokines such as IL-5, IL-17, CCL5, or MIP-1ß. Therefore, we examined the role of MIP-2 in AD. Downregulation of MIP-2 attenuated the clinical symptoms associated with AD induced by DNCB in BALB/c mice (Figure 4A). Atopic dermatitis increased the serum IgE level, the amount of histamine released, and ß-hexosaminidase activity in an MIP-2-dependent manner (Figure 4B). Atopic dermatitis increased the expression levels of SOCS1, TGaseII, COX-2, histone deacetylase 3 (HDAC3), and MCP1 in BALB/c mouse in an MIP-2-dependent manner (Figure 4C). Roles of TGaseII (Eom et al., 2014a), SOCS1 (Noh et al., 2017), COX-2 (Kwon et al., 2015), and HDAC3 (Kim et al., 2012; Eom et al., 2014b) in allergic inflammation, such as anaphylaxis, have been previously reported. Also, SOCS1 can suppress the immune modulatory function of MSCs by inhibiting the production of NO (Zhang et al., 2014). Downregulation of MIP-2 prevented DNCB from inducing interactions of Fc𝜀RIβ with HDAC3, Lyn, and SOCS1 (Figure 4C). Therefore, AD and anaphylaxis display common molecular features. Atopic dermatitis induced epidermal hyperplasia and lymphocyte infiltration. It also increased the numbers of degranulated MCs in an MIP-2-dependent manner (Figure 5). Immunohistochemical staining showed increased expression of MIP-2 by AD (Figure 5). Therefore, MIP-2 might mediate the atopic effect induced by DNCB.

### SOCS1, a Potential Target of MIP2-Regulated miR-122a-5p, Is Necessary for DNCB-Induced Atopic Dermatitis

**FIGURE 6:**
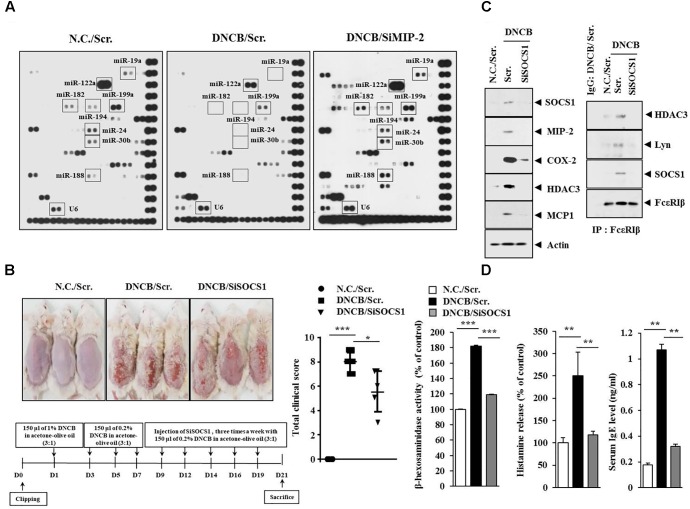
Suppressor of cytokine signaling 1 (SOCS1) is necessary for DNCB-induced AD. **(A)** Skin tissue lysates of BALB/c mice of each experimental group were subjected to miRNA array analysis. **(B)** Shows the experimental time line to determine the effect of MIP-2 on AD (lower). Upper figure shows the effect of SOCS1 on clinical symptoms associated with AD. ^∗^*p* < 0.05; ^∗∗∗^*p* < 0.0005. **(C)** Tissue lysates from the mice of each experimental group were subjected to western blot or immunoprecipitation by employing the indicated antibody (2 μg/ml). Tissue lysates from BALB/c mouse injected with scr. following the induction of AD was also immunoprecipitated with isotype-matched IgG antibody (2 μg/ml). **(D)** Tissue lysates from the mice of each experimental group were subjected to ß-hexosaminidase activity assays (left). Sera of BALB/c mice were employed for the determination of the amount of histamine released (middle) and IgE level (right). ^∗∗^*p* < 0.005; ^∗∗∗^*p* < 0.0005.

**FIGURE 7:**
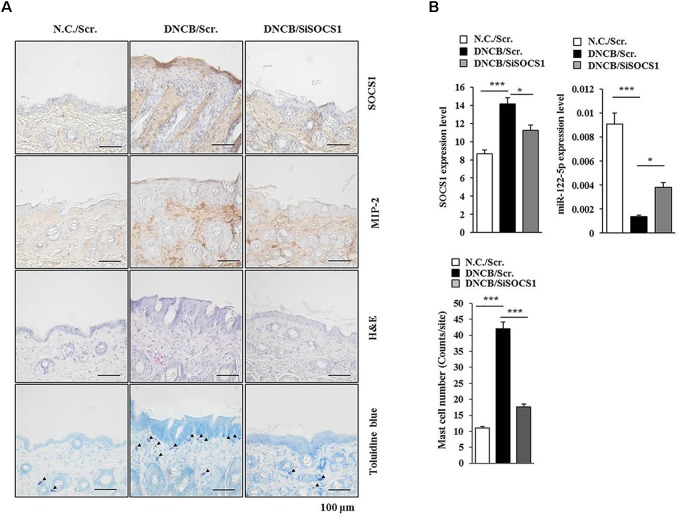
SOCS1 regulates the expression of miR-122a-5p and MIP-2. **(A)** Immunohistochemical staining by employing the indicated antibody was performed. Scale bar represents 10 μm. Skin tissues of BALB/c mice of each experimental group were isolated at the time of sacrifice and were also subjected to H&E staining. Toluidine blue staining was performed to determine the number of degranulated mast cells. Closed triangle represents degranulated mast cells. **(B)** Tissue lysates from mouse of each experimental group were subjected to qRT-PCR analysis. ^∗^*p* < 0.05; ^∗∗∗^*p* < 0.0005. The number of degranulated mast cells was also determined. Closed triangle represents degranulated mast cells. Scale bars represent 100 μm.

The roles of miRNAs in allergic inflammation have been reported as 18, 21. We hypothesized that MIP-2 would mediate AD by regulating miRNAs. For this, a miRNA array analysis was performed using skin tissue lysates of BALB/c mice. Additionally, miRNAs, such as miR-19a, miR-122a-5p, miR-182, miR-24, miR-30b, miR-188, and miR-199a, were downregulated by AD but upregulated by the downregulation of MIP-2 in BALB/c mouse under AD (Figure 6A). TargetScan analysis predicted that the binding of miR-122a-5p to the 3′ UTR of SOCS1 and miR-122a-5p would decrease luciferase activity associated with 3′ UTR of SOCS1 in rat basophilic leukemia (RBL2H3) cells (Noh et al., 2017). Since miR-122a-5p, downregulated by AD, was predicted to target SOCS1 in AD, we hypothesized that SOCS1 would be necessary for AD. The downregulation of SOCS1 attenuated the clinical symptoms associated with AD in BALB/c mice (Figure 6B). Atopic dermatitis increased the expression levels of MIP-2, COX-2, HDAC3, and MCP1 in BALB/c mice in a SOCS1-dependent manner (Figure 6C). Expression of COX-2 is known to be increased by house dust mite extract in Nc/Nga mouse model of AD (Karuppagounder et al., 2015). The downregulation of SOCS1 prevented AD from inducing interactions of Fc𝜀RIβ with HDAC3, Lyn, and SOCS1 in BALB/c mice (Figure 6C). It also prevented AD from increasing ß-hexosaminidase activity, serum IgE level, and the amount of histamine released in BALB/c mice (Figure 6D). Immunohistochemical staining showed increased expression levels of SOCS1 and MIP-2 by AD in BALB/c mouse (Figure 7A). The downregulation of SOCS1 prevented AD from inducing epidermal hyperplasia and lymphocytes infiltration (Figure 7A). It also increased the expression of miR-122a-5p in qRT-PCR analysis (Figure 7B). Therefore, miR-122a-5p and SOCS1 might form a negative feedback loop. The downregulation of SOCS1 prevented AD from increasing the number of degranulated MCs (Figures 7A,B). Taken together, these results suggest that SOCS1 might mediate AD.

### miR-122a-5p Mimic Attenuates Atopic Dermatitis by Regulating the Expression of Hallmarks of Allergic Inflammation Such as SOCS1

**FIGURE 8:**
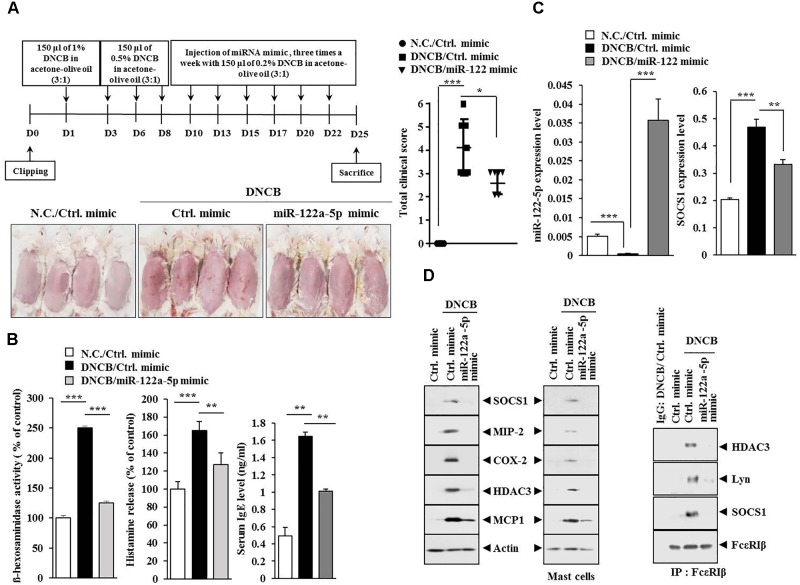
miR-122a-5p mimic inhibits DNCB-induced AD. **(A)** Shows the experimental time line to determine the effect of miR-122a-5p mimic on AD (upper). Lower figure shows the effect of miR-122a-5p mimic on clinical symptoms associated with AD. ^∗^*p* < 0.05; ^∗∗∗^*p* < 0.0005. **(B)** Tissue lysates from the mice of each experimental group were subjected to ß-hexosaminidase activity assays (left). Sera of BALB/c mice were employed for the determination of histamine release (middle) and IgE level (right). ^∗∗^*p* < 0.005; ^∗∗∗^*p* < 0.0005. **(C)** Tissue lysates from the mice of each experimental group were subjected to qRT-PCR analysis. ^∗∗^*p* < 0.005; ^∗∗∗^*p* < 0.0005. **(D)** Tissue lysates from the mice of each experimental group were subjected to western blot analysis (upper) or immunoprecipitation (lower) by employing the indicated antibody (2 μg/ml). Mast cell lysates from the skin tissue of the mice of each experimental group were also subjected to western blot analysis (upper right). Tissue lysates from BALB/c mouse injected with control (ctrl.) mimic following the induction of AD by DNCB were immunoprecipitated with isotype-matched IgG antibody (2 μg/ml).

**FIGURE 9:**
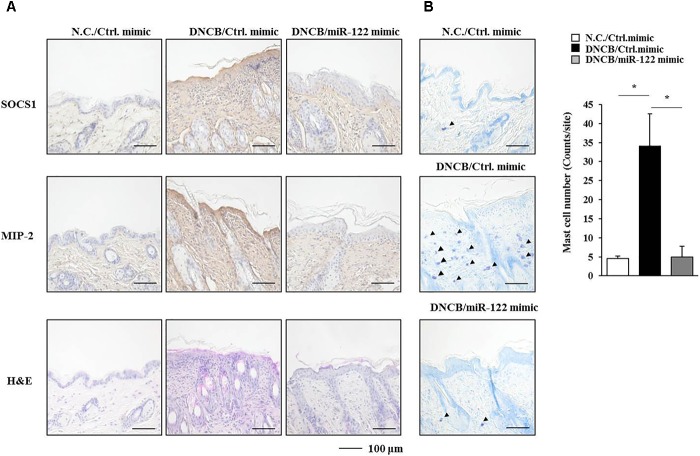
miR-122a-5p mimic negatively regulates the expression of SOCS1, MIP-2, and the number of degranulated mast cells. **(A)** Immunohistochemical staining by employing the indicated antibody was performed. Scale bar represents 100 μm. H&E staining was also performed. **(B)** Skin tissues of BALB/c mice of each experimental group were isolated at the time of sacrifice and were subjected to toluidine blue staining to determine the number of degranulated mast cells. Closed triangle represents degranulated mast cells. Scale bar represents 100 μm. ^∗^*p* < 0.05.

Since the downregulation of SOCS1 increased the expression of miR-122a-5p (Figure 7B), the effect of miR-122a-5p, a potential regulator of SOCS1, on AD was examined. The miR-122a-5p mimic attenuated the clinical symptoms associated with AD in BALB/c mice (Figure 8A). It also prevented DNCB from increasing ß-hexosaminidase activity, serum IgE level, and the amount of histamine released in BALB/c mice (Figure 8B). The miR-122a-5p mimic prevented DNCB from increasing the expression of SOCS1 at the transcriptional level (Figure 8C). It also prevented DNCB from increasing the expression levels of SOCS1, MIP-2, COX-2, HDAC3, MCP1 in skin tissue (Figure 8D, upper left), and MCs isolated from the skin tissue of BALB/c mouse (Figure 8D, upper right). The miR-122a-5p mimic prevented DNCB from inducing interactions of Fc𝜀RIβ with HDAC3, Lyn, and SOCS1 in the skin tissue of BALB/c mice (Figure 8D, lower). Immunohistochemical staining showed that miR-122a-5p mimic prevented DNCB from increasing the expression levels of SOCS1 and MIP-2 (Figure 9A). The miR-122a-5p mimic prevented DNCB from inducing epidermal hyperplasia and lymphocytes infiltration (Figure 9A). It also prevented DNCB from increasing the number of degranulated MCs in BALB/c mice (Figure 9B). Taken together, these results suggest that miR-122a-5p can attenuate AD by regulating the expression of hallmarks of allergic inflammation such as SOCS1.

### CXCL13 Is Necessary for DNCB-Induced Atopic Dermatitis and Anaphylaxis

**FIGURE 10:**
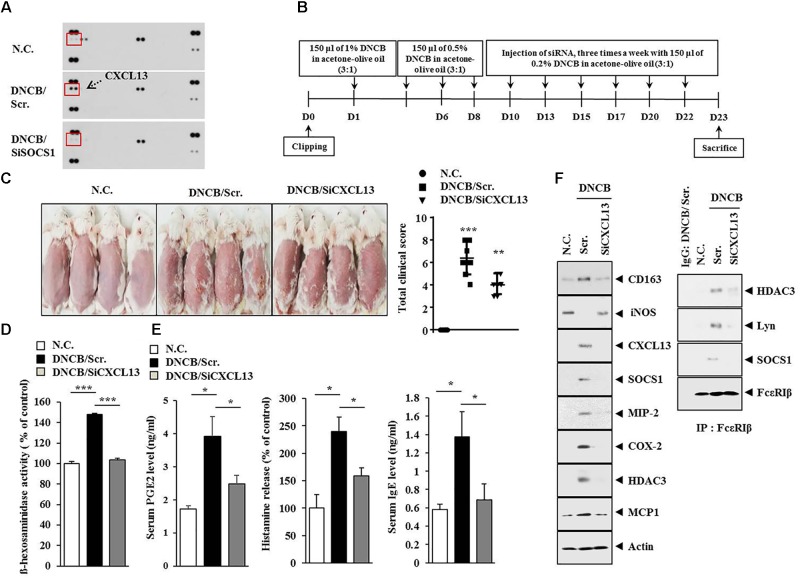
Chemokine (C-X-C motif) ligand 13 (CXCL13) is necessary for DNCB-induced AD. **(A)** Serum of BALB/c mouse of each experimental group was subjected to cytokine array analyses. Serum was obtained at the time of sacrifice. **(B)** Shows the experimental time line to determine the effect of CXCL13 on AD. (**C**) At the indicated day after the induction of AD, clinical scores of BALB/c mice of each experimental group were determined. ^∗∗^*p* < 0.005; ^∗∗∗^*p* < 0.0005. **(D)** Tissue lysates from the mice of each experimental group were subjected to ß-hexosaminidase activity assays. ^∗∗∗^*p* < 0.0005. **(E)** Sera of mice of each experimental group were employed for the determination of PGE2 level, IgE level, and the amount of histamine released. ^∗^*p* < 0.05. **(F)** Tissue lysates from the mice of each experimental group were subjected to western blot analysis and immunoprecipitation by employing the indicated antibody (2 μg/ml).

**FIGURE 11:**
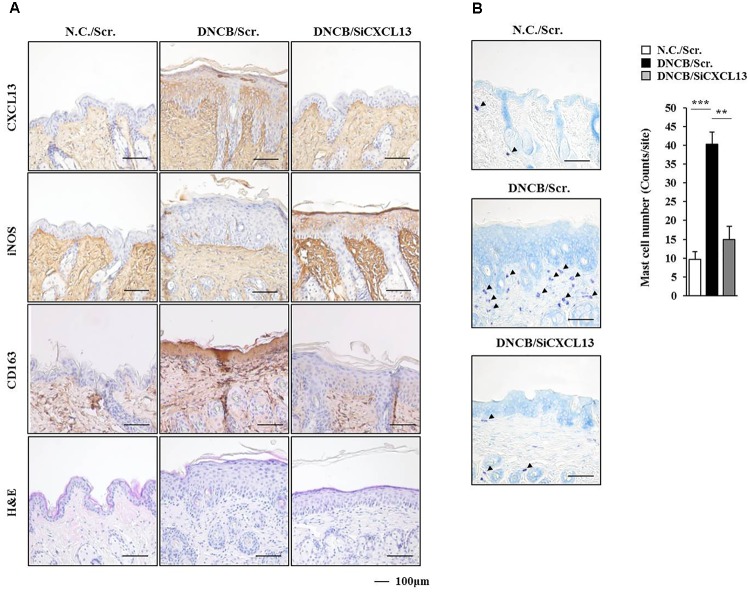
DNCB-induced AD regulates the expression of markers of activated macrophages. **(A)** Immunohistochemical staining by employing the indicated antibody was performed. Scale bar represents 100 μm. Skin tissues of BALB/c mice of each experimental group were isolated at the time of sacrifice and were also subjected to H&E staining. **(B)** Toluidine blue staining was performed to determine the number of degranulated mast cells. Closed triangle represents degranulated mast cells. ^∗∗^*p* < 0.005; ^∗∗∗^*p* < 0.0005.

**FIGURE 12:**
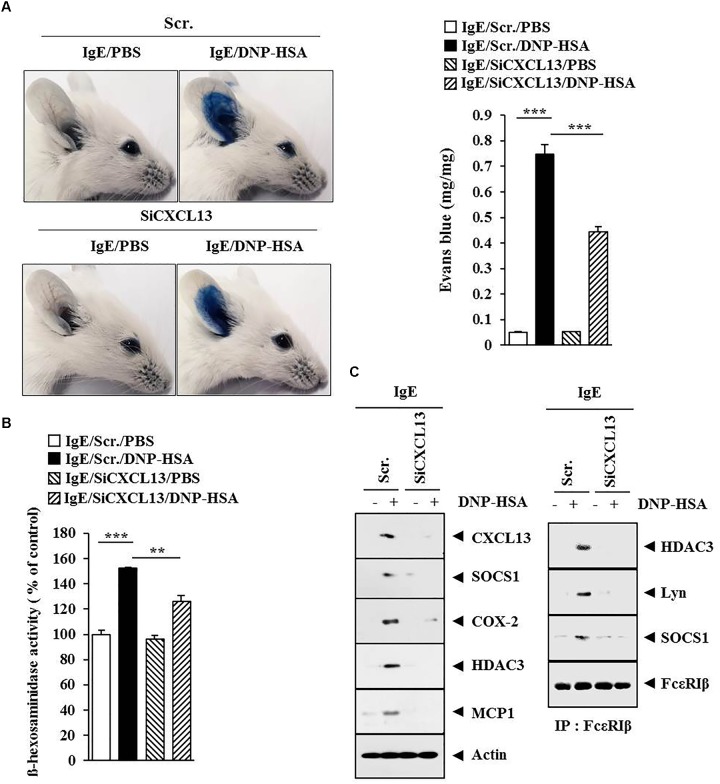
CXCL13 mediates passive cutaneous anaphylaxis. **(A)** Passive cutaneous anaphylaxis was performed as described. After 1 h of Evans blue solution injection, the dye was eluted from the ear in 700 μl of formamide at 63°C. The absorbance was measured at 620 nm. Representative images from four animals of each experimental group are shown. ^∗∗∗^*p* < 0.0005. **(B)** Ear tissue lysates from BALB/c mouse of each experimental group were subjected to β-hexosaminidase activity assay. ^∗∗^*p* < 0.005; ^∗∗∗^*p* < 0.0005. **(C)** Same as **(B)** except that western blot and immunoprecipitation were performed.

Since SOCS1 mediated AD (Figure 6B), we attempted to identify cytokines/chemokines regulated by SOCS1. For this, cytokine array analysis was performed by employing the sera of BALB/c mice under AD without or with siSOCS1 injection. Atopic dermatitis increased the expression of CXCL13 in the sera of BALB/c mice in a SOCS1-dependent manner (Figure 10A). We next examined the effect of CXCL13 on AD (Figure 10B). The downregulation of CXCL13 attenuated the clinical symptoms associated with AD (Figure 10C), prevented DNCB from increasing ß-hexosaminidase activity (Figure 10D), and prevented DNCB from increasing serum PGE2 level, serum IgE level, and the amount of histamine released in BALB/c mouse (Figure 10E). Atopic dermatitis increased the expression levels of CD163, SOCS1, MIP-2, COX-2, HDAC3, COX-2, and MCP1 but decreased the expression of iNOS in a CXCL13-dependent manner (Figure 10F). The downregulation of CXCL13 prevented DNCB from inducing interactions of Fc𝜀RIß with HDAC3, Lyn, and SOCS1 (Figure 10F). Immunohistochemical staining showed that AD increased the expression of CD163 while decreasing the expression of iNOS in a CXCL13-dependent manner (Figure 11A). Atopic dermatitis increased the number of degranulated MCs in a CXCL13-dependent manner (Figure 11B). Inducible nitric oxide synthase and CD163 are markers of M2 and M1 macrophages, respectively. It is probable that AD is accompanied by the activation of M2 macrophages. Since AD displayed molecular features of anaphylaxis (Figure 4C), the effect of CXCL13 on PCA was also examined. The downregulation of CXCL13 prevented the antigen (DNP-HSA) from increasing vascular permeability (Figure 12A) and ß–hexosaminidase activity (Figure 12B) in a mouse model of PCA. The downregulation of CXCL13 prevented the antigen from increasing the expression of hallmarks of allergic inflammation and interactions of Fc𝜀RIß with HDAC3, Lyn, and SOCS1 (Figure 12C). Taken together, these results suggest that CXCL13 may mediate AD and anaphylaxis by regulating the activation of MCs along with macrophages.

### miR-122a-5p–SOCS1 Axis Regulates Th1/Th2 and Treg Response During Atopic Dermatitis

**FIGURE 13:**
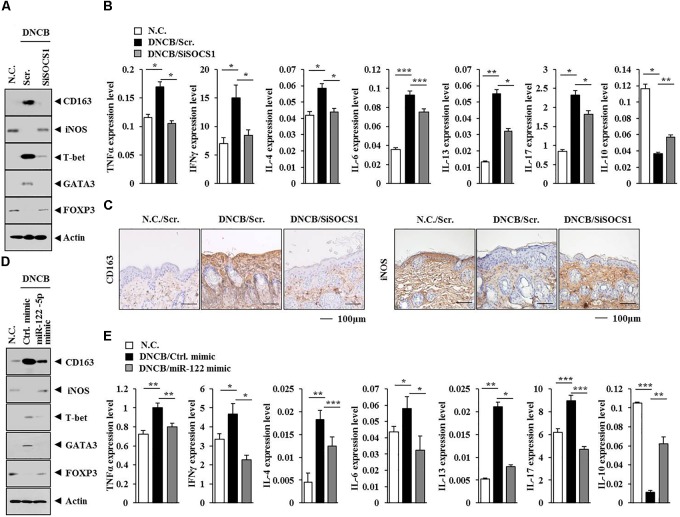
miR-122a-5p-SOCS1 negative feedback loop regulates TH1/TH2 and regulatory T (Treg) response during AD. **(A,D)** Tissue lysates from the mice of each experimental group were subjected to western blot analysis. **(C)** Skin tissues of BALB/c mice of each experimental group were subjected to immunohistochemical staining. Scale bar represents 100 μm. **(B,E)** Tissue lysates from the mice of each experimental group were subjected to qRT-PCR analysis.^∗^*p* < 0.05; ^∗∗^*p* < 0.005; ^∗∗∗^*p* < 0.0005.

The hUC-MSCs can reduce ovalbumin (OVA)-induced allergic inflammation. This effect might be mediated by regulatory T (Treg) cells (Kang et al., 2017). Human mesenchymal stem cells (HUMSCs) can reduce neutrophil airway inflammation by downregulating neutrophil chemokine production and by modulating T-cell responses (Hong et al., 2017). The AdMSCs are known to activate Treg cells. The activation of Treg cells involves secretion of PGE2 and TGF-ß by AdMSCs (Cho et al., 2014). The HUMSCs can also increase Treg cells via heme oxygenase-1 (HO-1) (Li et al., 2013). Also, GATA-3 and RORγt (transcription factors for Th2 and Th17 cell differentiation, respectively) determine the phenotype of asthmatic airway inflammation (Ano et al., 2013). It has been reported that OVA-exposed RORγt-overexpressing mice display higher expression of MIP-2 than wild type mice (Ano et al., 2013). These reports led us to hypothesize that AdMSCs might attenuate AD by regulating T cell responses. Atopic dermatitis increased the expression levels of T-bet and GATA-3 but decreased the expression of Foxp3 in BALB/c mouse, in an SOCS1-dependent manner (Figure 13A). The miR-122a-5p mimic prevented AD from regulating the expression of T-bet, GATA-3, and Foxp3 in BALB/c mouse (Figure 13D). Also, T-bet and GATA-3 are transcriptional factors of Th1 cells and Th2 cells, respectively. The protein Foxp3 is a specific transcriptional factor of Treg cells. Tumor necrosis factor-α and IFNγ are Th1 cytokines. The interleukins IL-4, IL-5, IL-6, and IL-13 are Th2 cytokines. In addition to this, IL-17 represents Th17 cells. Atopic dermatitis increased the expression levels of IL-4, IL-13, IL-6, IL-17, IFNγ, and TNFα but decreased the expression of IL-10 in BALB/c mouse, in an SOCS1-dependent manner (Figure 13B). The miR-122a-5p mimic prevented DNCB from increasing the expression of IL-4, IL-13, IL-6, IL-17, IFNγ, and TNFα (Figure 13E). Since AD increased the expression of IL-17, it would be necessary to examine the effect of AD on the expression of RORγT, a specific transcription factor of Th17 cells. Atopic dermatitis decreased the expression of IL-10 in BALB/c mouse in an SOCS1-dependent manner (Figure 13B). The miR-122a-5p mimic prevented DNCB from decreasing the expression of IL-10 (Figure 13E). Anti-IL-10 can block the expansion of Treg cells by myeloid-derived suppressor cells, and IL-10 induces Treg response and attenuates rheumatoid inflammation (Park et al., 2018). Atopic dermatitis, in an SOCS1-dependent manner, decreased the expression of iNOS while increasing the expression of CD163 in BALB/c mouse (Figure 13A). Immunohistochemical staining showed that SOCS1 was necessary for the expression regulation of CD163 and iNOS in mouse model of AD (Figure 13C). The miR-122a-5p mimic prevented AD from regulating the expression of iNOS and CD163 in BALB/c mouse (Figure 13D). It is probable that IL-10 might regulate the expression of CD163 and iNOS in BALB/c mice. Taken together, these results suggest that AD can induce Th1 and Th2 response while suppressing the Treg response in a SOCS1-dependent manner.

### Mast Cell Activation of Macrophages Occurs in Atopic Dermatitis

**FIGURE 14:**
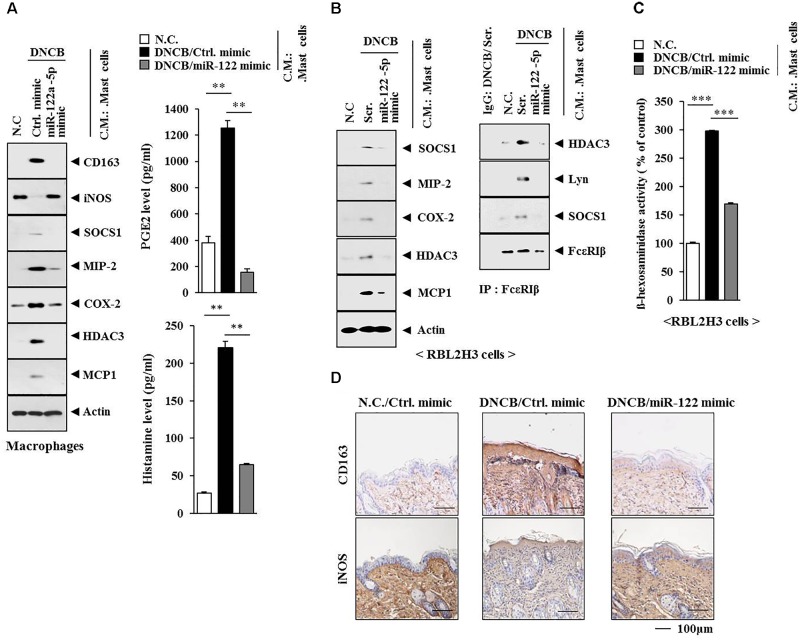
Mast cell activation of macrophages occurs in AD. **(A)** Skin mast cells were isolated from BALB/c mice of each experimental group as described. The conditioned medium of mast cells isolated from BALB/c mouse of each experimental group was added to lung macrophages for 8 h, followed by western blot analysis (left). The conditioned medium of mast cells isolated from BALB/c mouse of each experimental group was subjected to PGE2 level assays and histamine release assays (right). ^∗∗^*p* < 0.005. C.M., conditioned medium. **(B)** Same as **(A)** except that rat basophilic leukemia cells, equivalent of mouse mast cells, were employed. Western blot and immunoprecipitation were performed. Tissue lysates from BALB/c mouse injected with ctrl. mimic following the induction of AD by DNCB were immunoprecipitated with isotype-matched IgG antibody (2 μg/ml). **(C)** Same as **(B)** except that ß-hexosaminidase activity assays were performed. ^∗∗∗^*p* < 0.0005. **(D)** Skin tissues of BALB/c mice of each experimental group were subjected to immunohistochemical staining. Scale bar represents 100 μm.

Since AD was accompanied by the activation of macrophages (Figures 13A,C), we examined the possibility of cellular interactions between MCs and macrophages in AD. We hypothesized that soluble factors of MCs might activate macrophages. When the conditioned medium of MCs isolated from an AD-induced BALB/c mouse was added to macrophages isolated from a BALB/c mouse, it increased the expression levels of CD163 and hallmarks of allergic inflammation while decreasing the expression of iNOS in macrophages (Figure 14A). The conditioned medium of MCs isolated from an AD-induced BALB/c mouse showed higher levels of PGE2 and histamine released than the conditioned medium of MCs isolated from a BALB/c mouse or MCs isolated from an AD-induced BALB/c mouse injected with miR-122a-5p mimic (Figure 14A, right). When the conditioned medium of MCs isolated from an AD-induced BALB/c mouse was added to RBL2H3 cells, it increased the expression levels of hallmarks of allergic inflammation, induced interactions of Fc𝜀RIß with HDAC3, SOCS1, and Lyn (Figure 14B), and increased ß-hexosaminidase activity (Figure 14C). However, miR-122a-5p mimic prevented the conditioned medium of AD-activated MCs from activating macrophages and inducing features of AD (Figures 14A–C). Immunohistochemical staining showed that miR-122a-5p mimic prevented AD from regulating the expression of CD163 and iNOS in a BALB/c mouse (Figure 14D).

These results indicate that there are cellular interactions during AD. Further study is needed to identify cytokines/chemokines that mediate cellular interactions involving MCs and macrophages.

### Conditioned Medium of AdMSCs Inhibits Features of Allergic Inflammation

**FIGURE 15:**
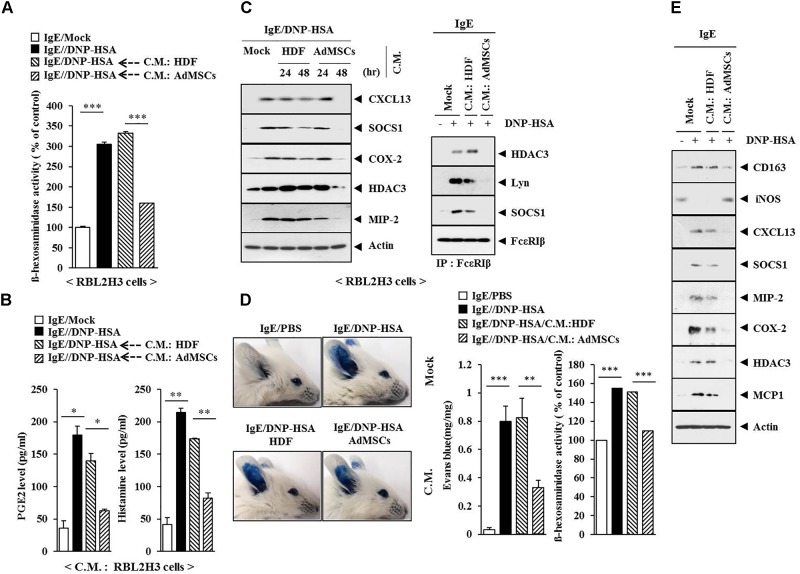
The conditioned medium of AdMSCs inhibits the features of allergic inflammation. **(A)** The IgE (100 ng/ml)-sensitized RBL2H3 cells were treated without or with the conditioned medium of HDFs or AdMSCs for 48 h, followed by stimulation with DNP-HSA (100 ng/ml) for 1 h. The ß-hexosaminidase activity assays were performed. ^∗∗∗^*p* < 0.0005. **(B)** Same as **(A)** except that PGE2 level and the amount of histamine released were determined by using the conditioned medium of RBL2H3 cells. ^∗^*p* < 0.05; ^∗∗^*p* < 0.005. **(C)** The IgE (100 ng/ml)-sensitized RBL2H3 cells were treated without or with the conditioned medium of HDFs or AdMSCs for the indicated time, followed by stimulation with DNP-HSA (100 ng/ml) for 1 h. Western blot was performed. For immunoprecipitation, lysates from RBL2H3 cells treated without or with conditioned medium for 48 h were employed. **(D)** BALB/c mice were given an intradermal injection of IgE (0.5 μg/kg) and intravenous injection of the conditioned medium of HDFs or AdMSCs (each at 200 μl/mouse). Representative images from four animals of each experimental group are shown. Ear tissue lysates from BALB/c mouse of each experimental group were subjected to β-hexosaminidase activity assays. ^∗∗^*p* < 0.005; ^∗∗∗^*p* < 0.0005. **(E)** Ear tissue lysates were subjected to western blot analysis.

Coculture experiments have shown that AdMSCs can attenuate AD by regulating MC degranulation and B cell function (Shin et al., 2017). This led us to hypothesize that soluble factors in AdMSCs might attenuate AD. The conditioned medium of AdMSCs, but not that of HDFs, prevented the antigen from increasing ß-hexosaminidase activity in RBL2H3 cells (Figure 15A). It also prevented the antigen from increasing the PGE2 level and the amount of histamine in the conditioned medium of RBL2H3 cells (Figure 15B). The conditioned medium of AdMSCs, but not that of HDFs, prevented the antigen from increasing the expression levels of hallmarks of allergic inflammation. It also prevented the antigen from inducing interactions of Fc𝜀RIß with HDAC3, SOCS1, and Lyn in RBL2H3 cells (Figure 15C). The conditioned medium of AdMSCs, but not that of HDFs, prevented the antigen from increasing vascular permeability and ß–hexosaminidase activity (Figure 15D). It also prevented the antigen from increasing the expression of hallmarks of allergic inflammation and CD163 while preventing the antigen from decreasing the expression of iNOS in a mouse model of PCA (Figure 15E). The conditioned medium of AdMSCs displayed higher expression levels of various cytokines, such as MCP1, CCL5, IL-6, and IL-8 than those seen in the conditioned medium of HDFs (Supplementary Figure S1A). The conditioned medium of AdMSCs, but not the conditioned medium of HDFs, prevented the antigen from increasing the expression of cytokines such as IL-7, MCP1, CXCL12, IL-27, and macrophage colony stimulating factor (M-CSF) in IgE-sensitized RBL2H3 cells (Supplementary Figure S1B). Thus, secreted cytokines/chemokines in the conditioned medium of AdMSCs might have acted as negative regulators of AD.

## Discussion

Our results showed that AdMSCs prevented AD from increasing the serum PGE2 level. The prostaglandin PGE2 can induce IL-22 from T cells through its receptors such as the prostanoid receptors EP2 and EP4 (Robb et al., 2018). Selective deletion of EP4 in T cells limits atopic-like skin inflammation (Robb et al., 2018). The interleukin IL-22 plays important pathogenic roles in the initiation and development of AD, in part through the induction of keratinocyte production of type 2 cytokines and activation of the gastrin-releasing peptide (GRP)/GRPR pathway (Lou et al., 2017). However, the effect of AdMSCs on AD and the mechanism of anti-atopic effect by AdMSCs have not been investigated extensively.

Mast cell activation involves increased expression of CCL5 (Oskeritzian et al., 2015; Du et al., 2016). The Asian AD phenotype presents increased hyperplasia, parakeratosis, higher TH17 activation, and a strong TH2 component (Noda et al., 2015). Allergic response to grass pollen involves increased expression of MIP-1β (Leaker et al., 2017). The interleukins IL-17 and IL-5 are known to contribute to pathologic changes in skin structure and barrier functions as well as immune dysregulation in AD (Dong et al., 2016; Purushothaman et al., 2018). Additionally, IL-17 and IL-5 represent TH17 cytokine and TH2 cytokine, respectively. The high molecular weight form of hyaluronic acid (HA) can reduce skin lesions in Nc/Nga mice treated with DNFB and decrease the expression levels of MIP-2, Sprr-2a, and serum IgE (Kim et al., 2008). We showed that expression levels of cytokines, such as IL-5, IL-17, CCL5, MIP-1ß (CCL4), and MIP-2, were increased by DNCB and downregulated by AdMSCs, but not by HDFs, in a mouse model of AD. In comparison with cytokines, such as IL-5, IL-17, CCL5, and MIP-1ß, the functional role of MIP-2 in AD has not been studied extensively yet.

The miRNA precursor miR-146a can inhibit chronic skin inflammation by decreasing the expression levels of IFN-γ, CCL5, and CCL8 in the skin (Rebane et al., 2014). Keratinocytes of patients with AD show increased IFN-γ-induced apoptosis compared to keratinocytes from healthy subjects. They also show higher expression levels of CCL5 and CCL8 than healthy subjects (Rebane et al., 2012). Also, miR-143 can suppress IL-13 activity and inflammation by targeting IL-13Rα1 in epidermal keratinocytes (Zeng et al., 2016). In addition to this, miR-155 is over-expressed in patients with AD. It might be involved in AD pathogenesis by modulating differentiation and function of Th17 cells (Ma et al., 2015).

The miRNA miR-122a-5p that is negatively regulated by MIP-2 in a mouse model of AD is known to form a negative feedback loop with SOCS1, and it regulates allergic inflammation such as anaphylaxis (Noh et al., 2017). Allergen-induced airway inflammation is regulated by the upregulation of SOCS1 (Dharajiya et al., 2009). Numbers of circulating CD4 ^+^CXCR5 ^+^ICOS ^+^PD-1 ^+^ T_FH_-like cells are significantly increased in children with AD when compared with healthy controls (Szabó et al., 2017). Chemokine (C-X-C motif) receptor 5 (CXCR5) is a receptor for CXCL13. The stimulation of Fc𝜀RI increases the numbers of macrophages expressing histamine receptor 1 (Novak et al., 2013). The numbers of CD163 (+) cells in lesional skin of AD patients are larger than those seen in normal skin (Han et al., 2015). These reports suggest the involvement of activated macrophages in AD. Our results showed that AD increased the expression of CD163 while decreasing the expression of iNOS in a SOCS1-dependent manner. We showed that CXCL13, an SOCS1-regulated cytokine, was necessary for the expression regulation of CD163 and iNOS.

The inhibition of allergic reactions is accompanied by increased numbers of CD4 (+) Foxp3 (+) T cells (Han et al., 2015). This suggests that Treg cells play an inhibitory role in AD. Our results showed that AD decreased the expression of Foxp3 in a SOCS1-dependent manner. Murine AdMSCs reduce allergic airway responsiveness, expression levels of lgE and IL-17F (*P* < 0.001), as well as increase the serum levels of Foxp3, and the percentage of CD4 (+) CD25 (+) Foxp3 (+) Treg cells in the spleen (Dai et al., 2018). It will be necessary to examine the effects of AdMSCs on the activation of Treg responses in a mouse model of AD.

Stem cell therapy has shown therapeutic benefits in immune modulation and tissue remodeling (van den Akker et al., 2013). However, the survival time of implanted cells is short. This greatly impairs the therapeutic effect of MSCs (Chimenti et al., 2010). In addition, transplantation of MSCs into normal tissues may cause tumor formation (Sell, 2010).

Recent reports have suggested that therapeutic effects of stem cells are due to factors such as cytokines, microRNAs, and exosomes, which are secreted by stem cells (Kay et al., 2017; Li et al., 2018; Palomares et al., 2018). The AdMSCs expressing inducible protein-10 (IP-10) reduce melanoma tumor growth and lung metastasis (Mirzaei et al., 2018). The conditioned medium of AdMSCs can promote endometrial cancer growth and invasion (Chu et al., 2018). These reports suggest that the conditioned medium of AdMSCs may attenuate AD by regulating cellular interactions. Our results showed that the conditioned medium of AdMSCs inhibited features of allergic inflammations.

Exosomes of AdMSCs can regulate intercellular communication in pathological processes of the skin (Liu et al., 2018). The AdMSCs-exosomes can significantly decrease the expression levels of various inflammatory cytokines such as IL-4, IL-23, IL-31, and TNF-α in AD skin lesions of Nc/Nga mice (Cho et al., 2018). Further studies are needed to examine the effect of GW4869, an inhibitor of exosomes formation, on anti-allergic effects by the conditioned medium of AdMSCs.

In summary, AdMSCs can attenuate AD by targeting MIP-2, which in turn regulates the expression of miR-122a-5p, a negative regulator of SOCS1. In addition to this, SOCS1 regulates the expression of CXCL13. Also, SOCS1 and CXCL13 are necessary for the activation of macrophages and regulation of T cells responses during AD. Experiments employing the conditioned medium demonstrate that there are cellular interactions during AD and the role of soluble factors of AdMSCs in regulating allergic inflammation. Thus, employing targets of AdMSCs such as MIP-2 might offer a valuable strategy to develop anti-atopic therapy.

## Author Contributions

MK, S-HL, and DJ designed the study. MK, YKw, YP, YKi, and S-HL performed the experiments and analyzed the data. HJ, H-KL, and DJ planned the projects and supervised the experiments. DJ wrote the manuscript.

## Conflict of Interest Statement

S-HL and H-KL were employed by EHL-BIO company. The remaining authors declare that the research was conducted in the absence of any commercial or financial relationships that could be construed as a potential conflict of interest.

## Footnotes

1 http://www.targetscan.org/

2 http://pictar.mdc-berlin.de/

3 http://mirdb.org/

## Supplementary Material

Figure S1 (A) Cytokine array analysis employing the conditioned medium of AdMSCs and HDFs was performed. **(B)** The IgE (100 ng/ml)-sensitized RBL2H3 cells were treated without or with the conditioned medium of HDFs or AdMSCs for 48 h, followed by stimulation with DNP-HSA (100 ng/ml) for 1 h. The conditioned medium of IgE-sensitized RBL2H3 cells without antigen stimulation (DNP-HSA) was also subjected to cytokine array analysis.
