# Supplementary figures and images for: Human Adipose Tissue-Derived Mesenchymal Stem Cells Attenuate Atopic Dermatitis by Regulating the Expression of MIP-2, miR-122a-SOCS1 Axis, and Th1/Th2 Responses
Source: Front Pharmacol. 2018 Nov 6;9:1175. doi: 10.3389/fphar.2018.01175 (PMC6232252; doi:10.3389/fphar.2018.01175)

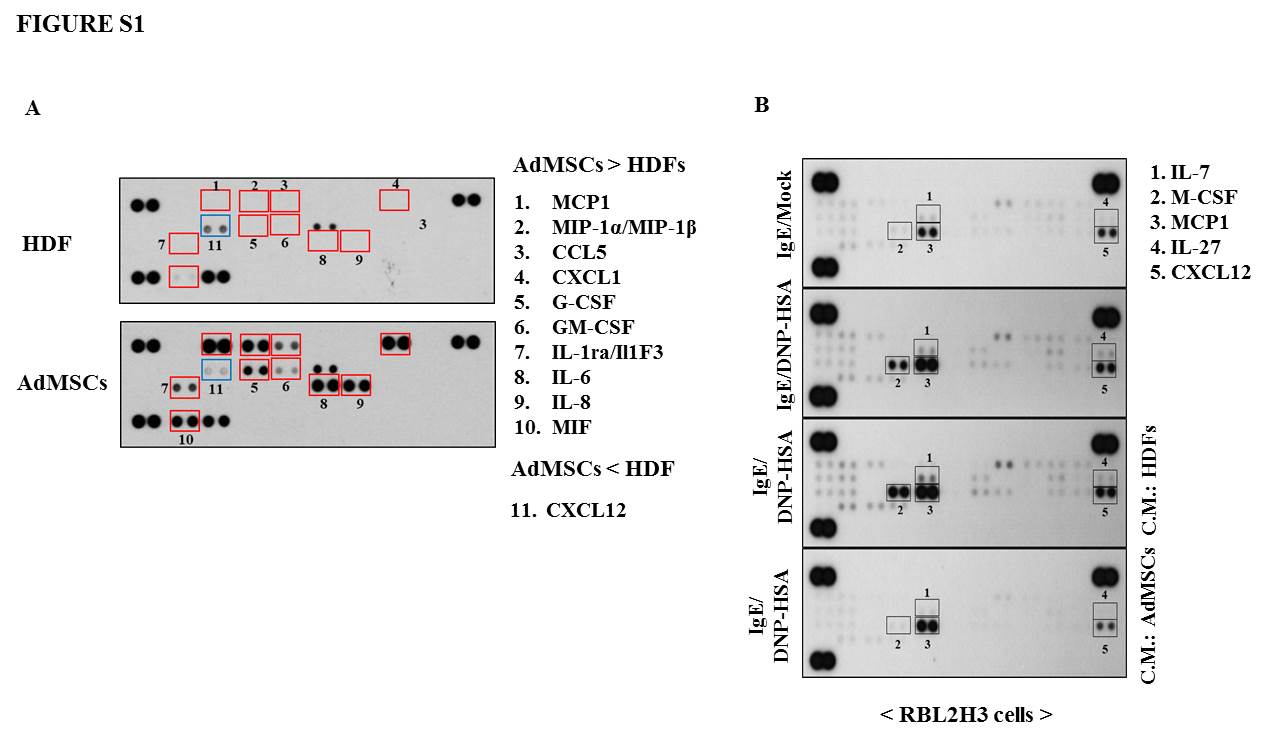

Supplement: Figure S1 — (A) Cytokine array analysis employing the conditioned medium of AdMSCs and HDFs was performed. (B) The IgE (100 ng/ml)-sensitized RBL2H3 cells were treated without or with the conditioned medium of HDFs or AdMSCs for 48 h, followed by stimulation with DNP-HSA (100 ng/ml) for 1 h. The conditioned medium of IgE-sensitized RBL2H3 cells without antigen stimulation (DNP-HSA) was also subjected to cytokine array analysis. [file Image_1.jpeg]
